# Bioprinted Three-Dimensional Cell-Laden Hydrogels to Evaluate Adipocyte-Breast Cancer Cell Interactions

**DOI:** 10.3390/gels6010010

**Published:** 2020-03-24

**Authors:** Sarah Chaji, Jenna Al-Saleh, Cheryl T. Gomillion

**Affiliations:** School of Chemical, Materials, & Biomedical Engineering, University of Georgia, Athens, GA 30602, USA

**Keywords:** 3D hydrogels, 3D bioprinting, breast cancer, bioink, cell-laden, adipocytes, stem cells

## Abstract

Three-dimensional (3D) bioprinting, although still in its infancy as a fabrication tool, has the potential to effectively mimic many biological environments. Cell-laden 3D printed structures have demonstrated to be an improvement from the widely used monolayer platforms, largely because of recapitulation of native tissue architecture with the 3D structures. Thus, 3D *in vitro* models have been increasingly investigated for improved modeling of cell and disease systems, such as for breast cancer. In the present work, multicellular cell-laden hydrogels comprised of adipocytes and breast cancer cells were bioprinted and evaluated. An ideal bioink of 3:2 5% alginate was determined to mimic the tissue stiffness observed in a physiological breast cancer tumor environment. Rheological characterization and degradation studies were performed to verify the stability of the artificial breast hydrogel environment. It was found that both the breast cancer cells and adipocytes remained viable directly after printing and throughout the 10-day culture period within the printed hydrogels. Direct printing of the cells in co-culture resulted in morphology changes and variations in cell localization within printed structures. Overall, the feasibility of efficiently fabricating multicellular cell-laden bioprinted models of the breast tumor microenvironment was established.

## 1. Introduction

For many years, breast cancer has paved the way as the second leading cause of cancer death in women in the Unites States [1,2,3]. It is estimated that approximately 1 in every 8 women and 1 in every 1000 men in the United States will develop invasive breast cancer [4]. These risks are even more detrimental for those who are overweight/obese postmenopausal women [5,6]. In addition, the percentage of total fat volume in the breast can range from 7 to 56%, on average [7], indicating that adipose tissue makes up a large portion of the breast environment and as such, should be considered when studying breast tumor cell behavior. With the likelihood of obtaining breast cancer in one’s lifetime progressively increasing, the importance for properly studying this disease has become vital. For decades, breast cancer has been evaluated using two-dimensional (2D) monolayer platforms despite 2D platforms lacking both the physiological and chemical cues represented in the *in vivo* microenvironment [8,9,10].

Mimicking breast cancer is challenging due to the various roles that different cells play. Structurally, the breast is comprised of adipose tissue, lobes, lobules, and ducts, which make up the fatty, fibrous, and granular tissues of the breast [7]. When combining these associated cell types (i.e., adipocytes, fibroblasts, mammary epithelial cells, etc.) in a 2D platform they have shown to behave differently than in a three-dimensional (3D) platform, with 3D platforms most resembling *in vivo* conditions and outcomes [11]. With appropriately designed 3D models, comparable physiological conditions can be achieved without the use of animal models; however, sufficient replication of native tissue is required for appropriate evaluation when studying a disease. For example, breast cancer has been linked to body mass index due to the influence of adipocytes and their secreted products [12,13]. These secreted factors influence the upregulation of cancer production and cancer associated adipocytes [14,15]. Co-culturing adipocytes with cancer cells has demonstrated an upregulation of osteopontin, TNF-α, IL-6, IL-1B, leptin, and adiponectin [16]. This upregulation of factors has resulted in increased breast cancer proliferation, including metastatic behavior. These hostile cells promote the production of cancer associated adipocytes, which then continue the deadly cycle [17,18]. Currently, however, there is no approach that allows scientists to accurately study these phenomena.

To overcome this limitation of suitable model platforms, 3D *in vitro* models such as formed using scaffolds, microfluidic devices, and 3D bioprinting have been developed to study breast cancer metastasis. At present, 3D bioprinting has emerged as a popular method for fabricating 3D tissue structures, as 3D bioprinters are advantageous due to their biomimicry capabilities [19]. Using 3D bioprinting techniques, the biological and biochemical components are able to be precisely positioned in a controlled manner using layer-by-layer approaches. An innovation such as this has allowed others to use 3D bioprinting to fabricate numerous forms of mammalian tissue, tumors, and even organs [20,21,22,23]. Researchers working on breast cancer specifically found that through 3D bioprinting, complex tumors and bone matrices can be replicated [24]. These bioprinted models have proven to be representative of the breast cancer tumor with the development of good vascularization and a complex multicellular environment which can be used for *in vitro* drug screening [25]. With this method, the biocompatibility, immunocompatibility, cell stability, and overall model structure can be better ensured, making 3D bioprinting an attractive option for fabricating 3D tissue models. 

Thus, we have determined this technique as ideal for printing the 3D breast cancer tumor environment, where resulting printed tissue structures would support cellular growth, cell-cell interactions and vascularization, while maintaining a supporting structure composed of comparable extracellular matrix proteins. Overall, 3D bioprinters come in three different forms: inkjet, extrusion-based, and laser-assisted [19]. The limitation of these bioprinters is typically the cost and size. Existing 3D bioprinters tend to be large and can cost anywhere from $10,000 to over $200,000 [26], restricting widespread application of this technology. Therefore, this proof-of-concept study was intended to show the feasibility of 3D bioprinting the breast cancer microenvironment using a small low-cost 3D extrusion printer modified as an extrusion-based 3D bioprinter. This was done by combining cells with a hydrogel mimicking the stiffness of breast cancer tissue in pursuit of producing a functional bioink.

## 2. Results and Discussion

### 2.1. Hydrogel Bioink Screening

Hydrogels are optimal materials for bioinks because they can provide an easy way to facilitate a biocompatible 3D encapsulation structure, providing a permeable porosity that allows the exchange of media, nutrients, and waste associated with cell proliferation and essential to tissue engineering applications [27]. Based on the physical properties of hydrogels, these materials are also well-suited for mimicking soft tissue, as is the goal of this work; thus, hydrogel biomaterials were selected for printing. Here, preliminary screening of various hydrogel materials identified an optimal composite of alginate and gelatin for bioprinting.

**Figure 1:**
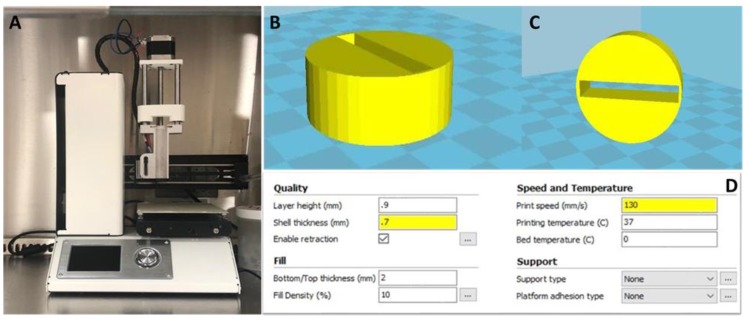
(**A**) Extrusion-based 3D printer modified and used for bioprinting applications. (**B**) AutoCAD design for 3D bioprinter. Cylinder sitting on x-axis design as printed. (**C**) Cylinder rotated 90° off the x-axis. (**D**) Basic settings calibrated for cell-laden 3D hydrogel bioprinting.

A commercially-available extrusion-based 3D printer with attached syringe holder (Figure 1) was used for all sample preparation in this work (Tissue Scribe Gen. 3, 3D Cultures). Screening of various materials to identify an optimal bioink basis was performed. As shown in Table 1, only the alginate/gelatin mixture being crosslinked in 0.05 M calcium chloride was able to deliver the desired criteria, thus an alginate/gelatin composite was used for subsequent experiments.

### 2.2. Characterization of Bioprinted 3D Hydrogels

With mammary tissue being largely comprised of adipose tissue, which is characterized as a soft tissue, evaluating adipocyte-breast cancer cell interactions is essential to mimicking the mammary carcinoma environment [28]. Not only is maintaining the appropriate cell-cell interactions important, but the stiffness of any fabricated microenvironment will play a crucial role as well. While the elastic moduli for normal glandular and mammary adipose tissue have been measured to be between 2 and 66 kPa, [29,30] cancerous breast tissues have demonstrated increased stiffness up to seven times greater, [31] with breast carcinoma having a mean shear stiffness 418% higher than the surrounding breast tissue [30]. These variations in stiffness of the breast cancer microenvironment have also been correlated to the specific cancer subtype, with triple negative and HER2+ tumors being stiffer [32]. Thus, to mimic breast tissue and cancerous breast tissue, the ideal tumor-mimicking hydrogel would have an elastic modulus value between 0.5 kPa and 25 kPa [29,30].

#### 2.2.1. Rheological Characterization

**Figure 2:**
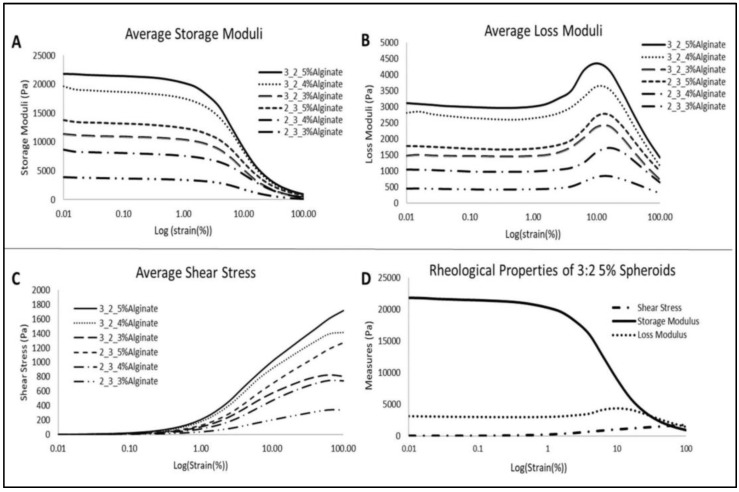
Average storage (**A**) and loss (**B**) moduli were measured for each 3D hydrogel composite to determine the best material for printing cell-laden hydrogels. Storage moduli and loss moduli were highest for the 3:2 5% alginate hydrogels. (**C**) Shear stress plot of the evaluated hydrogels show the 3:2 5% alginate hydrogels with the most resistance to shear stress. (**D**) Rheological analysis of the selected hydrogel composition. Storage modulus and loss modulus values show more elastic than viscous behavior. Shear stress values indicate that the 3D hydrogels cannot sustain large amounts of shear force.

Six different compositions of alginate and gelatin were used to prepare 3D hydrogels, consisting of 2:3, or 3:2 alginate to gelatin ratios, prepared using either 3, 4 or 5% alginate. Analysis of the rheological properties for each of these composites showed that in the viscoelastic region, the storage modulus was higher than the loss modulus for all samples (Figure 2A,B). The yield point for all samples was around 1% strain, after which the internal integrity of the samples changed. Changes after 1% strain are not considered given that these samples did not undergo more than 1% strain during cell culture experiments. As shown by the obtained plots for both loss and storage modulus (Figure 2A,B), the loss and storage modulus were proportional to alginate concentration, where the loss and storage moduli increased as the alginate concentration increased. As shown in Figure 2C, the shear stress for each composite is also proportional to the alginate concentration. The 3:2 5% alginate to gelatin 3D hydrogels exhibited the highest storage and loss moduli, in addition to the highest resistance to shear stress. Shear stress any higher than approximately 205 Pa causes the structure of our hydrogels to change. Given that we use static culture, however, this did not pose a risk to our studies. The solution with the highest storage and loss modulus, 3:2 5% alginate to gelatin, was chosen for further studies and used for bioprinting experiments. The full rheological profile of the selected 3:2 5% alginate composite is shown in Figure 2D. The storage modulus for the 3:2 5% alginate to gelatin hydrogels is much greater than its loss modulus. Rheological data specifically showed that this concentration, when crosslinked for 15 minutes in 0.05M calcium chloride, provided tumor-mimicking hydrogels with suitable soft tissue stiffness properties, as reported for native breast cancer tissues.

#### 2.2.2. Bioprinted 3D Hydrogel Degradation and Morphology

**Figure 3:**
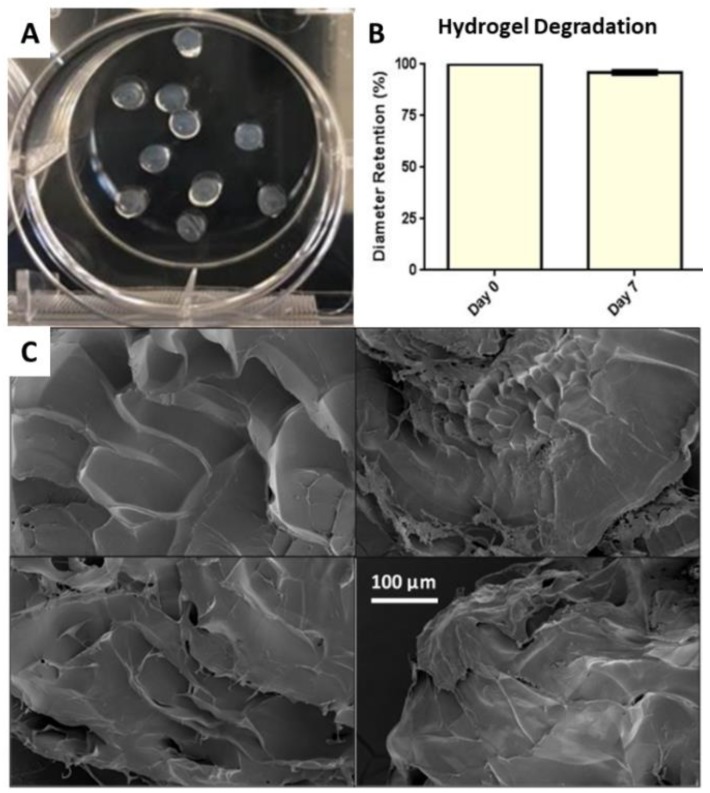
(**A**) Representative image of bioprinted hydrogels post-crosslinking. (**B**) Diameter retention of 3:2 5% alginate 3D hydrogels at Day 0 and Day 7. (**C**) Scanning electron microscopy images show the rough surface of the printed structures with minimal porosity.

Three-dimensional hydrogels formed post printing are shown in Figure 3A. The average diameter of the 3:2 5% alginate to gelatin hydrogels before degradation were 2117 ± 16.05 µm and 2214 ± 19.55 µm for Batch 1 and Batch 2, respectively. After incubation for the degradation study, the average 3D hydrogel diameter was 2009 ± 14.50 µm and 2146 ± 11.32 µm, respectively. After 7 days of incubation for degradation analysis, the 3:2 5% alginate to gelatin hydrogels retained approximately 96% of their diameter (Figure 3B). Scanning electron microscopy (SEM) was used to visualize the surface morphology of the printed hydrogels. As shown in Figure 3C, the surface of hydrogels maintained a rough texture due to cross-linking behavior. Minimal porosity was observed.

As demonstrated by SEM images, the bioprinted 3D hydrogels are not completely porous. The roughness of the hydrogels surface suggests these hydrogels could support good cell attachment, but this was not confirmed or essential in this work. Future studies could include z-stack imaging of the 3D hydrogels to confirm this and also methods for introducing greater porosity into the structure, while maintaining cell viability. This selected hydrogel composite was suitable as a bioink with optimal printability (i.e., extrusion from the syringe and needle) demonstrated. In addition, the alginate/gelatin composite provided adequate structural support for the 3D hydrogel environment, while allowing for sufficient degradation in order to encourage cell proliferation.

Overall, it was essential for the bioprinted 3D hydrogels to maintain their structure long enough for the culturing period with minimal degradation. Based on results of the rheological and degradation analysis, the concentration of 3:2 5% alginate to gelatin was selected for use to fabricate cell-laden 3D hydrogels. With a higher storage modulus observed as compared to the loss modulus in the linear viscoelastic region, the analyses provide evidence that our materials are more elastic than viscous. Increased storage modulus can be associated with increased stiffness, which is most likely due to increased cross-linked chemical bonds [33,34]. This was evidenced by the 3:2 5% alginate to gelatin hydrogels, as the increased alginate concentration allows for an increased number of cross-links between polymer molecules. Increased cross-links between alginate molecules yields a stiffer 3D hydrogel that is more resistant to degradation, as evidenced in our degradation studies. Further, increased chemical cross-links provide more structural integrity, which affirmed the selection of the 3:2 5% alginate hydrogel for further studies.

### 2.3. Preliminary Screening of Culture Medium

For preparing cell-laden 3D hydrogels, in addition to selection of an optimal bioink, it was equally important to determine optimal culturing parameters for the selected cells. MCF-7 breast cancer cells and adipose-derived stromal cells (ADSCs) pre-differentiated to mature adipocytes were the cell types used for these experiments. Structurally, mature adipocytes have lipid-filled cell cytoplasms, with vast unilocular lipid droplets within the cell. In addition, these mature adipocytes are capable of secreting factors such as tumor necrosis factor- alpha (TNF-α), interleukin-6 (IL-6), and leptin [35,36,37]. Through early trials of the printing process, the fragile nature of these intracellular lipids was observed with the harsh stresses of bioprinting resulting in bursting or rupture of the adipocytes. In order to overcome this limitation and maintain the secretory potential of lipid-filled mature adipocytes, alterations to the printer and printing settings were conducted, as well as adjustments to the desired time and duration of pre-differentiation *in vitro*. 

Differentiation media was used to achieve adipogenesis, or conversion of ADSCs to mature adipocytes. However, the dedifferentiation of adipocytes is a prevalent phenomenon that has been observed in response to physical cues, temperature fluctuations or other chemical cues [38]. Thus, to avoid this issue, a preliminary evaluation of culture media was performed. During the preliminary evaluation, all 2D cultures and cell-laden 3D hydrogels were cultured in differentiation media. The pre-differentiated ADSCs showed no signs of dedifferentiation, while the MCF-7s unexpectedly displayed abnormal signs of lipid presence, visible with Oil Red O and Nile Red staining (data not shown). This presence and formation of lipids within cancer cells has been previously shown as a response to high stress levels [39], thus indicating our need to optimize the culture conditions. Others have successfully studied the different forms of cancer behavior throughout the printing process and observed similar results [25,40]. Subsequently, we then used a formulation of adipogenic maintenance media (AM) instead of differentiation media to maintain adipogenic phenotype for the ADSCs without yielding abnormal effects for the cancer cells in co-culture. A comparison of the AM to control DMEM-Complete medium (DC) was performed.

#### 2.3.1. Two-Dimensional Cell Viability and Metabolic Activity

**Figure 4:**
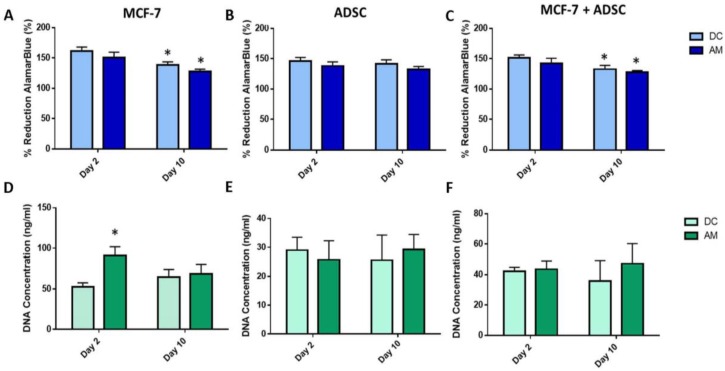
Cancer cell and adipocyte cell metabolic activity (as indicated by percent reduction alamarBlue^®^ reagent) in monoculture (**A**,**B**) and co-culture (**C**) in 2D monolayer culture. DNA content as measured using PicoGreen^®^ assay for MCF-7 cancer cells (**D**) and adipocytes (**E**) in monoculture and co-culture (**F**). Asterisks (*) indicate a statistically significant difference (*p* < 0.05).

Metabolic activity, determined by the alamarBlue^®^ assay showed a statistically significant decrease in metabolic activity (*p* < 0.05) for both media types from Day 2 to 10 for MCF-7s (Figure 4A) and no significant change in ADSC metabolic activity (Figure 4B). Measurement of DNA concentration using PicoGreen^®^ assay showed a significant increase in DNA concentration for MCF-7s at Day 2 for the AM in comparison to DC (Figure 4D). For ADSCs grown in 2D, there was no significant difference in DNA concentration observed for any media formulation (Figure 4E). Co-culture of the ADSCs and MCF-7 cells together in 2D culture showed a significant decrease in metabolic activity from Day 2 to Day 10 for both the DC and AM media (*p* < 0.05). However, there were no significant differences observed in DNA concentration for the co-culture samples using either media type (Figure 4F).

#### 2.3.2. Two-Dimensional Adipogenic Potential

**Figure 5:**
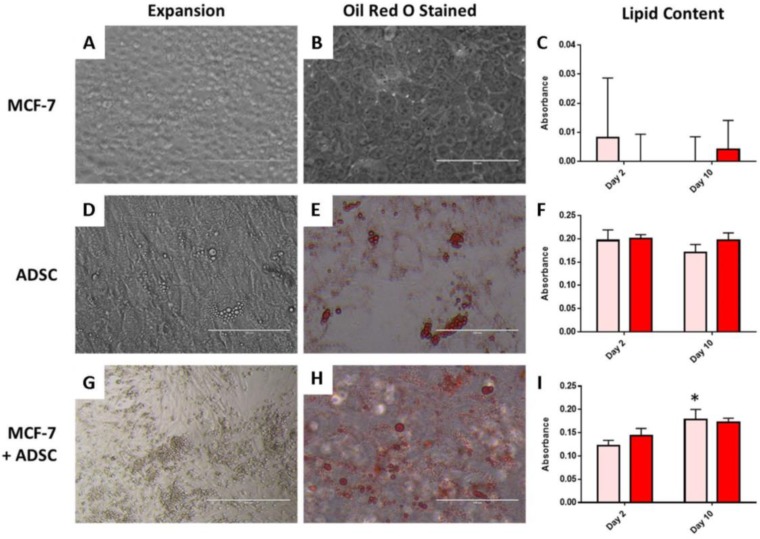
Expansion of (**A**) MCF-7 cells, (**D**) ADSCs and (**G**) MCF-7 and ADSCs co-cultured after 10 days in 2D culture. (**B**,**E**,**H**) show representative images of cells stained with Oil Red O to detect intracellular lipid produced following culture in adipogenic maintenance medium. The lipid content with respective absorbance values for each condition are shown in (**C**,**F**,**I**). Scale bar = 400 µm.

Representative cell morphology for MCF-7 and pre-differentiated ADSCs are shown in Figure 5A,D, respectively. The MCF-7 cells maintain their epithelial-like morphology, while ADSCs have visible lipid droplets present, indicating their differentiation to mature adipocytes prior to seeding and printing using the established differentiation media. ORO staining was used to confirm the presence of lipid droplets within the adipocytes. As shown in Figure 5B, no lipid is detected in the MCF-7 cells when stained with ORO, as expected. Lipid droplets are apparent, as shown in Figure 5E, for ADSCs differentiated to adipocytes. Quantification of the ORO destained solution showed little to no lipid for MCF-7 cells cultured with either media type (Figure 5C). There were greater amounts of lipid content measured for the ADSCs, however, no significant difference was observed for ADSCs using either DC or AM media (Figure 5F). The 2D co-culture of ADSCs and MCF-7 cells showed that the ADSCs were still able to differentiate in the presence of the cancer cells, as indicated by lipids visible via ORO staining (Figure 5H) and confirmed by quantification of the destained solution. For the co-cultured cells, from significantly higher lipid content (p<0.05) was observed from Day 2 to Day 10 for the DC media (Figure 5I). 

The maintenance media demonstrated the ability to prevent dedifferentiation of the differentiated ADSCs without provoking a stress response within the cancer cells in 2D. Overall, the use of DMEM-Complete and adipogenic maintenance media displayed no long-term significant difference for cell viability or adipogenic potential in 2D culture. 

### 2.4. Evaluation of 3D Bioprinted Cell-Laden Hydrogels

Cancer cells can react differently based on the stiffness of the environment in which they are placed [40]. Thus, identifying a hydrogel composition with the appropriate stiffness was essential for this study. Viability of both cell types in 3D and the adipogenic potential of ADSCs in 3D was assessed using monocultures to identify optimal culturing conditions. Cells were then co-cultured for final observation.

#### 2.4.1. Assessment of 3D Cell-Laden Hydrogel Monocultures

**Figure 6:**
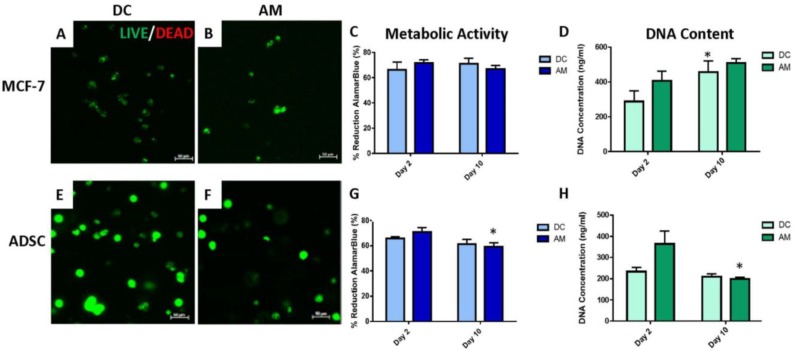
3D monoculture assesment of MCF-7 and ADSC cell-laden hydrogels in DMEM-Complete and adipogenic maintenance media. (**A**,**B**,**E**,**F**) LIVE/DEAD^®^ images. (**C**,**D**,**G**,**H**) AlamarBlue^®^ and PicoGreen^®^ quantification of metabolic activity and cell viability. Scale bar = 50 µm.

LIVE/DEAD^®^ analysis showed that both the MCF-7s and predifferentiated ADSCs remained viable after 10 days post-printing within 3D hydrogels when cultured with both media types (Figure 6A,B,E,F). AlamarBlue^®^ analysis to assess metabolic activity in the 3D platform showed no significant difference in metabolic activity for MCF-7s for either media type for 3D hydrogels (Figure 6C). The metabolic activity of the ADSCs was significantly less (*p* < 0.05) at Day 10 than at Day 2 when cultured in the AM media (Figure 6G). PicoGreen^®^ assay to quantify DNA content showed a significant increase (*p* < 0.05) in DNA concentration for the MCF-7 cells cultured in DC media from Day 2 to Day 10 (Figure 6D). ADSCs, on the other hand, had a significant decrease (*p* < 0.05) in DNA concentration from Day 2 to Day 10 when cultured in AM media (Figure 6H).

**Figure 7:**
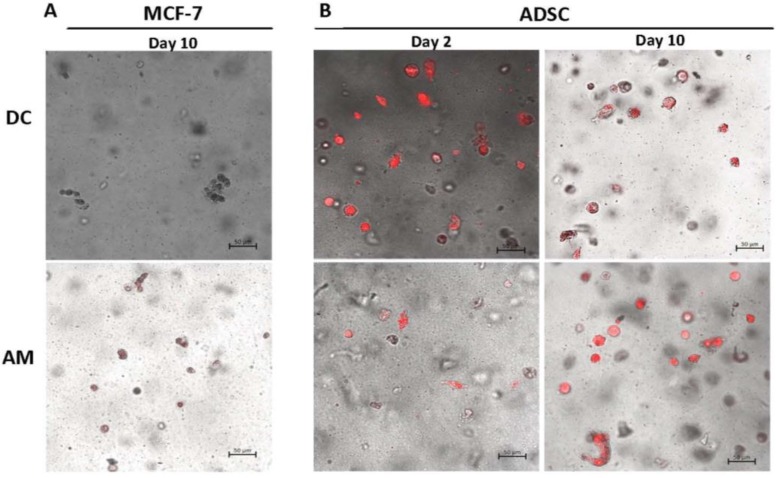
3D monoculture Nile Red staining of lipids for (**A**) MCF-7 and (**B**) ADSC with DC and AM. Scale bar = 50 µm.

Qualitative analysis of lipid presence for the 3D samples was conducted using Nile Red staining. Visualization of stained hydrogels revealed no staining in MCF-7s with either media type (Figure 7A), as was expected. Staining of the ADSCs showed the presence of lipids in ADSCs at both Day 2 and Day 10 when cultured in both media types.

The LIVE/DEAD^®^ assay demonstrated that both the pre-differentiated adipocytes and the MCF-7s were able to remain viable with the selected printing alterations, indicating that the adjusted printing parameters were not too aggressive for cell viability. AlamarBlue^®^ and PicoGreen^®^ revealed the 3:2 5% alginate to gelatin hydrogel worked as a suitable bioink by maintaining cell viability for 10 days post-printing. The cells were able to proliferate and extract nutrients from the media through the hydrogel. Nile Red staining showed that lipids remained intact in the 3D platform. Based on the Oil Red O staining it is expected that the lipid concentration in 3D increased over time as well, although this was not quantifiable with the Nile Red stain used. When comparing cells cultured in 2D or in 3D cell-laden hydrogels post-printing with either media type, it was determined that the amount of lipid formed in differentiated ADSCs was maintained at a comparable level (when quantified with ORO) with just DMEM-Complete post-printing, thus the maintenance media was not essential. Cells were subsequently cultured only using DMEM-Complete for subsequent analyses of hydrogels with co-cultured cells. 

#### 2.4.2. Assessment of 3D Cell-Laden Hydrogel Co-Cultures

**Figure 8:**
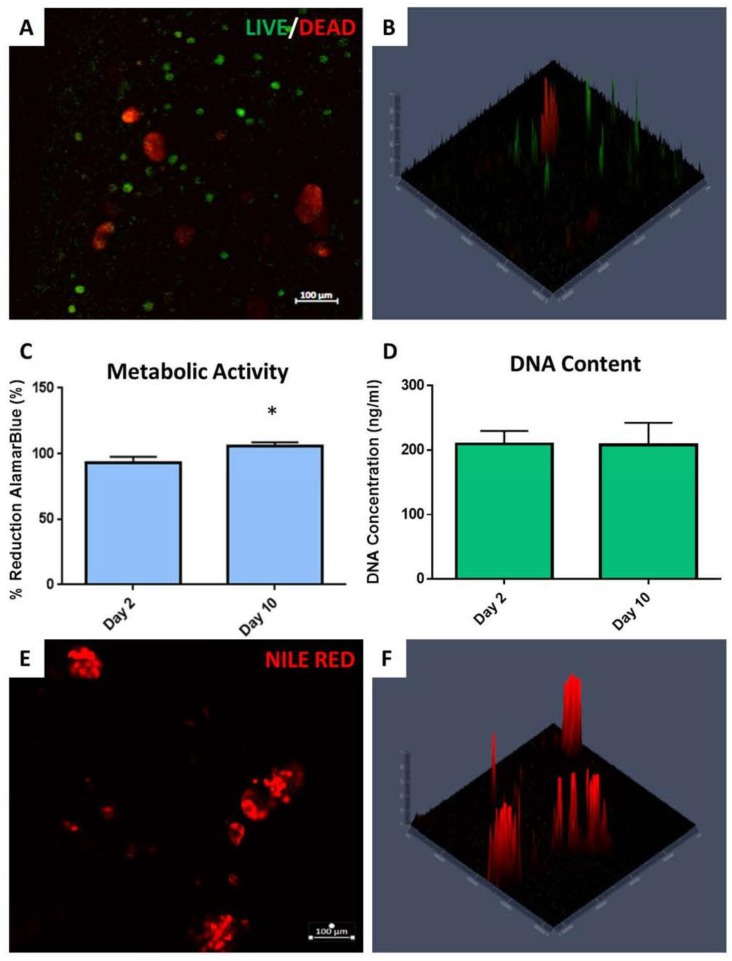
3D co-cultured cell-laden hydrogels. (**A**,**B**) LIVE/DEAD^®^ images with topography map. (**C**,**D**) AlamarBlue^®^ and PicoGreen^®^ quantification of metabolic activity and cell viability. (**E**,**F**) Nile Red staining of lipids with topography map showilng lipid distribution throughout.

LIVE/DEAD^®^ staining of cell -laden hydrogels with co-cultured MCF-7 and ADSCs showed that the majority of cells remained viable after 10 days (Figure 8A,B). Non-viable cells, stained red, or necrotic cells (shown yellow in the overlay image) tended to be clustered together. As shown in Figure 8B, the confocal imaging topography map for the stained samples showed that the red-stained cells extended deeper into the center of the hydrogel. AlamarBlue^®^ data indicated that a significant increase (*p* < 0.05) in metabolic activity at Day 10 in comparison to Day 2 (Figure 8C), however, the DNA concentration determined by PicoGreen^®^ assay showed no significant increase over time. Evaluation of adipogenic potential, as indicated qualitatively with Nile Red staining, showed significant lipid presence in 3D hydrogels with co-cultured MCF-7 and ADSCs at Day 10 (Figure 8E,F). The lipids in the 3D co-culture platform appeared more clustered together in comparison to the ADSCs alone shown in Figure 7B. Figure 8F shows the confocal imaging topography map of the lipids present in the co-culture environment, which reveals the depth of the lipids within the hydrogel structure.

With the 3D co-culture hydrogels containing both MCF-7 cells and pre-differentiated ADSCs, only DMEM-Complete was used as the media source for culture post-printing. The co-culture presented viable cells 10 days after printing, demonstrating survival of the cells under these printing conditions. The co-cultured cells had a tendency of moving closer to one another to form deep clusters within the innermost areas of the 3D structures. The cells within these clusters seemed to fluoresce both green and red, suggesting some necrotic behavior. This occurrence is not atypical however, for breast tumors, where hypoxic conditions within the interstitial spaces of the tumor can affect cell viability, [41] further demonstrating the physiological similarities of our bioprinted cell-laden hydrogels here and native breast cancer tissues. Even with LIVE/DEAD staining indicating some supposed necrotic behavior, there was an increase in DNA concentration and significant increase in total metabolic activity observed over time, indicating growth and activity of the cells during the culture period. The lipids within the 3D co-culture environment also tended to cluster closer to one another extending deeper into the 3D hydrogels. The ADSC hydrogels displayed a more isolated dispersion of lipids while the co-culture showed them forming a bundle, suggesting an effect by the MCF-7 cancer cells on adipocyte behavior in the co-cultured samples.

## 3. Conclusions

With this proof-of-concept work, we successfully demonstrated the use of a low-cost modified printer for bioprinting of cell-laden 3D hydrogels. We successfully recapitulated the 3D breast cancer tumor environment with intact adipocytes and breast cancer cells for evaluation. Thus, we have developed a platform with significant future clinical application. With modification, future application could entail bioprinting of a patient’s specific breast cancer cells in conjunction with their own adipocytes for a high-throughput screening technique for identifying breast cancer treatment options. Future work will entail optimization of printed structures for modeling proliferation, migration, and metastasis influenced by adipocytes to improve methods towards developing a breast cancer cure.

## 4. Materials and Methods 

### 4.1. Materials

Alginic acid sodium salt and porcine gelatin (300g Bloom) were obtained from Sigma-Aldrich (St. Louis, MO, USA). Calcium chloride dehydrate was obtained from Fisher (Fair Lawn, NJ, USA). Deionized MilliQ water (18 MΩ) was obtained from an in-house purification system. Adipose-derived stromal cells (ADSCs) and MCF-7 mammary adenocarcinoma cells (ER^+^, PR^+^) were obtained from the American Type Culture Collection (ATCC, Manassas, VA, USA).

### 4.2. Bioprinter Optimization

Physical alterations were made directly to the Tissue Scribe printer, including re-wiring of hardware to support hydrogel extrusion. Adjustments to the CURA software settings were also made to optimize this low-cost printer for printing cell-laden 3D hydrogels. For optimal and efficient printing of uniform 3D hydrogels, a cylindrical design was created in AutoCAD (Autodesk, 2018), as shown in Figure 1B,C. The AutoCAD image was converted to an STL file and imported into the printer’s CURA software for slicing.

CURA 15.04.2 consists of basic and advanced printing settings. The basic settings as follows: for quality, a layer height of 0.1 mm and shell thickness of 0.7 mm were used, with the enable reaction box selected. For the fill settings, a bottom/top thickness of 2 mm and fill density of 10% were used. A printing speed of 130 mm/s was used along with a printing temperature of 37 °C and a bed temperature of 0 °C. The filament was assigned a diameter of 2.85 mm with 100% flow, as shown in Figure 1D.

Advanced settings consisted of nozzle size of 1.2 mm, retraction with a speed of 40.0 mm/s and distance of 4.5 mm. The quality settings were set to initial layer thickness of 0.3 mm, initial layer line width of 100%, cut off object bottom of 0 mm, and dual extrusion overlap of 0 mm. The multiple speed options were set at a travel speed of 150 mm/s, bottom layer speed of 20 mm/s, infill speed of 2 mm/s, top/bottom speed of 2 mm/s, outer shell speed of 2 mm/s, and inner shell speed of 2 mm/s. The cool for the printer had a minimal layer time of 20 s and enable cooling fan was selected.

### 4.3. Hydrogel Bioink Screening 

Initial screening to identify optimal materials for printing 3D hydrogels with desired physical properties was performed. Six different polymer materials, agarose, chitosan, pectin, alginate, gelatin and a blend of alginate and gelatin, were tested using solvents and crosslinkers as denoted in Table 1. Formed 3D hydrogels were prepared using the bioprinter and stored at 37 ℃. Resulting 3D hydrogels were incubated for up to 14 days and observations were made regarding maintenance of structure, degradation and cell viability post-crosslinking.

#### 4.3.1. Three-Dimensional Hydrogel Fabrication and Characterization

Based on the preliminary screening, a composite blend of alginate and gelatin was identified as the optimal medium for 3D cell-laden hydrogel printing and used for all subsequent experiments. Composites consisting of various concentrations of alginate (ranging from 3‒5% *w*/*v*) and selected ratios of gelatin were tested to identify optimal crosslinking and extrusion properties for printing 3D hydrogels. Both the alginate and gelatin powders were first sterilized using ethylene oxide sterilization to decrease the chances of contamination while maintaining structural integrity of the hydrogels. All subsequent preparation and use of the hydrogel solution were carried out within a biosafety cabinet to protect the cell-laden from pathogens. 

To create both alginate and gelatin liquid solutions, the powders were dissolved in sterile deionized (DI) water for 2 hours at 40 °C while stirring at a medium speed on a stir plate. The 3D hydrogels initially tested were prepared using 3%, 4%, and 5% (*w*/*v*) alginate solutions that were mixed with a 50 mg/mL porcine gelatin solution, creating a 1:2, 2:1, 2:3, or 3:2 alginate to gelatin ratio mixture. Based on observations of crosslinking efficacy and degradation, the 1:2 and 2:1 composites were excluded from subsequent evaluation. Prior to printing, the solutions were warmed to 37 °C to support cell viability and ensure printability of the solution when extruded out of the syringe. The warmed hydrogel solution was printed into a 0.05 M calcium chloride crosslinking solution in a Petri dish and allowed to crosslink in the solution for 15 minutes. The 3D structures were rinsed with 1X Dulbecco’s Phosphate Buffered Saline solution (PBS) and samples were then characterized to assess their resulting physical properties. 

#### 4.3.2. Rheological Characterization

Rheological properties of the printed hydrogels were determined using an MCR 302 Anton Paar Rheometer (Anton Paar, Ashland, VA, USA). A two-plate system was used with a PP25/S plunger 25 mm in diameter in order to perform an amplitude sweep on each set of hydrogels. Six categories of hydrogels (2:3, or 3:2 alginate to gelatin ratios prepared using either 3, 4 or 5% alginate) were studied using rheology. For each category, four samples were measured, with each sample consisting of 40‒45 3D hydrogels for measurement. The angular frequency was held constant at 10 radians/s, and the amplitude deflection angle was changed in intervals from 0.1% until 100% deflection. The temperature was held at a constant 37 °C. Each sample was measured with a gap of 2 mm between the plates. The storage modulus, loss modulus and shear stress were all recorded for analysis.

#### 4.3.3. Three-Dimensional Hydrogel Degradation

Degradation of printed hydrogels was observed to confirm an optimal ratio of alginate to gelatin. Preliminary evaluation with non-sterile prepared hydrogels (using the previously described biomaterial combinations) helped determine the optimal ratio and concentration of alginate to gelatin, which was then evaluated using sterile prepared samples. For degradation studies, 10 hydrogel samples prepared using a 3:2 alginate to gelatin with 5% alginate, were printed and cross-linked in 0.05 M calcium chloride for 15 min. The samples were then rinsed and photographed on a fixed stage at a height of 11.5 cm. Post-imaging, all hydrogels were placed in 2 mL of PBS (Ca^2+^ and Mg^2+^-free) and incubated at 37 °C and 5% CO_2_ for 7 days. At the end of the 7-day period, the excess PBS was removed and samples were photographed again at the same height. Image J software (2018, National Institutes of Health) was used to calculate the hydrogel diameters and areas at both time points. Printed hydrogel weight before and after incubation was also recorded. This process was performed twice using different batches for both the non-sterile and sterile preparation to account for any batch-to-batch variability.

#### 4.3.4. Scanning Electron Microscopy

Sample hydrogels prepared using 3:2 alginate to gelatin with 5% alginate were fixed using 1 mL of formalin. Cell-laden hydrogel samples were then placed in PBS and lyophilized for 24 h. Dried samples were then gold-coated using a Leica EM ACE600 coater (Leica, Buffalo Grove, IL, USA). After coating was complete, a FEI Teneo field emission scanning electron microscope (FEI, Inc., Hillsboro, OR, USA) was used to capture images.

### 4.4. Cell Expansion and Seeding

MCF-7 cells, ranging from passage 7‒15, were grown to confluence. ADSCs from passage 8‒11 were grown to 95% confluence and then pre-differentiated into mature adipocytes for 7 days. Both MCF-7s and ADSCs were expanded using proliferation media consisting of low-glucose Dulbecco’s Modified Eagle Medium (DMEM), 10% fetal bovine serum (FBS), and 1% penicillin/streptomycin (P/S), denoted as DMEM-Complete. Pre-differentiation of ADSCs into adipocytes was performed using adipocyte differentiation media consisting of high-glucose DMEM, 10% FBS, 1% P/S, 3-isobutyl-1-methylxanthine (IBMX), 100 µM indomethacin, 10 µM rosiglitazone, 0.02% dexamethasone, and 0.1% insulin.

### 4.5. Preliminary Screening of Culture Medium

A preliminary evaluation of cells cultured with DMEM-Complete (DC) or adipogenic maintenance media (AM) was performed to identify the optimal culture medium for supporting both cancer cell viability and maintaining the adipogenic characteristics of predifferentiated ADSCs. ADSCs and MCF-7 cells were seeded separately in 2D monoculture or in a co-culture with both cell types in the same well. Adipogenic maintenance media consisted of low-glucose DMEM, 10% FBS, 0.1% insulin, 0.02% dexamethasone, and 1% P/S. Cells were seeded at a seeding density of 7.6 × 10^4^ cells/well for each condition, with a 50:50 ratio of MCF-7s to ADSCs used for co-culture conditions. Cells were cultured for 10 days and assessed at Day 2 and Day 10 of culture. 

### 4.6. Bioprinting of 3D Cell-laden Hydrogels

Cell-laden bioprinted hydrogels containing either adipocytes or MCF-7 cells were first assessed to confirm optimal media formulation for 3D cultures. Cells were seeded at a total density of 5 × 10^5^ cells/mL or 1 × 10^6^ cells/mL for each condition, with a 50:50 ratio of MCF-7s to ADSCs used for co-culture conditions. A total of five 3D hydrogels were printed directly into wells of 24-well Ultra-low Attachment plates (Corning, Tewksbury, MA, USA), and samples were cultured for 10 days with fresh media replaced in the wells every 2‒3 days. Following determination of optimal media formulation for 3D cultures, bioprinted hydrogels containing a co-culture of adipocytes and MCF-7 cells were also printed using the same methods. Assays of cell viability, metabolic activity and lipid content were performed at Day 2 and Day 10 of culture to evaluate efficacy of printing and culture methods. 

### 4.7. In Vitro Cell Viability and Metabolic Activity Assessment

Cell behavior was assessed for all 2D and 3D cultures after 2 and 10 days of seeding or printing. Cellular metabolic activity was assessed using alamarBlue^®^ assay (Pierce Biotechnology, Rockford, IL, USA), with sample absorbance measured using a Biotek 800TS microplate reader (Winooski, VT, USA) at 570 and 600 nm. DNA concentration was determined using the Quant-iT PicoGreen^®^ assay (Molecular Probes, Invitrogen, Eugene, OR, USA) according to the manufacturer’s protocol with slight modification. Briefly, a series of freezing and thawing cycles, in combination with a Cyquant Cell Lysis Buffer (Invitrogen, Eugene, OR, USA), was used to lyse the cells for DNA quantification. Hydrogels were dissociated using a 1:1 ratio of 0.2M sodium citrate solution and 1X cell lysis buffer. Cell viability was also qualitatively assessed for 3D cell-laden hydrogels using a LIVE/DEAD^®^ Viability/Cytotoxicity assay (Molecular Probes, Invitrogen, Eugene, OR, USA) according to manufacturer specifications. Images were collected using a Zeiss LSM 710 confocal microscope (Zeiss, Oberkochen, Germany) using the Zeiss AXIO Observer Z1 microscope stand. 

### 4.8. Adipogenic Potential 

Oil Red O (ORO) staining was performed to quantify lipids present in the 2D cultures. Cells were washed with PBS and fixed using 10% formalin. The wells were then washed with 60% isopropanol and allowed to dry. A stock solution of ORO powder (Sigma-Aldrich, St. Louis, MO, USA) dissolved in 100% isopropanol was used to prepare an ORO working reagent. The working reagent was added to the cell cultures and incubated for 10 minutes at room temperature with gentle shaking on a rocker for staining. Next, the wells were rinsed 4 times with DI water and images of the stained cells were captured. A volume of 500 µL of 100% isopropanol was added to each well and incubated for 15 minutes at room temperature for destaining. The absorbance of the ORO destained solution was measured at 500 nm using a Biotek 800TS microplate reader for quantifying lipid content. 

The adipogenic potential of the 3D cell-laden hydrogels was assessed qualitatively using Nile Red staining. A 1 µM working solution of Nile Red was created by dissolving Nile Red powder (MP Biomedicals, Solon, OH, USA) in small amounts of dimethyl sulfoxide (DMSO) and further diluted in 1X Hank’s Balanced Salt Solution (HBSS). The hydrogels were incubated in the working solution for 15 minutes at 37 ℃ and washed with PBS. Images were captured using the Zeiss LSM 710 confocal microscope at 552 nm.

### 4.9. Statistical Analysis

All statistical analyses were performed using GraphPad Prism 6^™^ (GraphPad Software, Inc.) Two-way ANOVA followed by Tukey post-tests for multiple comparisons were performed to determine statistical significance between individual sample groups with significance set at p < 0.05. Data are expressed as mean and standard deviation (SD).

## Figures and Tables

**Table 1:** Materials screened for 3D hydrogel formation.

Hydrogel Material	Solvent	Crosslinker	Structure Maintained (Y/N)	Significant Degradation Observed (Y/N)	Cells Viable After Crosslinking (Y/N)
Agarose	Deionized H_2_O	None/Cooled PBS	Y	Y	Y
Chitosan	1% Glacial Acetic Acid	0.5 N Sodium Hydroxide	Y	N	N
Pectin	Deionized H_2_O	1 M Calcium Chloride	N	Y	Y
Alginate	Deionized H_2_O	1 M Calcium Chloride	Y	Y	Y
Gelatin	Deionized H_2_O	None/Cooled PBS	Y	Y	Y
Alginate/Gelatin	Deionized H_2_O	0.05 M Calcium Chloride	Y	N	Y
